# Predicting kidney failure risk after acute kidney injury among people receiving nephrology clinic care

**DOI:** 10.1093/ndt/gfy294

**Published:** 2018-10-15

**Authors:** Simon Sawhney, Monica Beaulieu, Corri Black, Ognjenka Djurdjev, Gabriela Espino-Hernandez, Angharad Marks, David J McLernon, Zainab Sheriff, Adeera Levin

**Affiliations:** 1 Division of Applied Health Sciences, University of Aberdeen, Foresterhill, Aberdeen, UK; 2 Division of Nephrology, University of British Columbia, Vancouver, BC, Canada

**Keywords:** acute kidney injury, epidemiology, kidney failure, prediction, prognosis

## Abstract

**Background:**

Outcomes after acute kidney injury (AKI) are well described, but not for those already under nephrology clinic care. This is where discussions about kidney failure risk are commonplace. We evaluated whether the established kidney failure risk equation (KFRE) should account for previous AKI episodes when used in this setting.

**Methods:**

This observational cohort study included 7491 people referred for nephrology clinic care in British Columbia in 2003–09 followed to 2016. Predictors were previous Kidney Disease: Improving Global Outcomes–based AKI, age, sex, proteinuria, estimated glomerular filtration rate (eGFR) and renal diagnosis. Outcomes were 5-year kidney failure and death. We developed cause-specific Cox models (AKI versus no AKI) for kidney failure and death, stratified by eGFR (</≥30 mL/min/1.73 m^2^). We also compared prediction models comparing the 5-year KFRE with two refitted models, one with and one without AKI as a predictor.

**Results:**

AKI was associated with increased kidney failure (33.1% versus 26.3%) and death (23.8% versus 16.8%) (P  < 0.001). In Cox models, AKI was independently associated with increased kidney failure in those with an eGFR ≥30 mL/min/1.73 m^2^ {hazard ratio [HR] 1.35 [95% confidence interval (CI) 1.07–1.70]}, no increase in those with eGFR <30 mL/min/1.73 m^2^ ([HR 1.05 95% CI 0.91–1.21)] and increased mortality in both subgroups [respective HRs 1.89 (95% CI 1.56–2.30) and 1.43 (1.16–1.75)]. Incorporating AKI into a refitted kidney failure prediction model did not improve predictions on comparison of receiver operating characteristics (P = 0.16) or decision curve analysis. The original KFRE calibrated poorly in this setting, underpredicting risk.

**Conclusions:**

AKI carries a poorer long-term prognosis among those already under nephrology care. AKI may not alter kidney failure risk predictions, but the use of prediction models without appreciating the full impact of AKI, including increased mortality, would be simplistic. People with kidney diseases have risks beyond simply kidney failure. This complexity and variability of outcomes of individuals is important.

## INTRODUCTION

Acute kidney injury (AKI) is a serious condition [1] with established risk factors [age, chronic kidney disease (CKD) and vascular comorbidities] [2]. It is associatied with increased long- term renal replacement therapy (RRT) [3], mortality [4] and CKD progression even when post-AKI kidney function returns to ‘normal’ [5]. However, most publications focus on hospitalized patients cared for by generalists. There are limited data regarding the impact of episodes of AKI among people already under the care of nephrologists [6]. Those under the regular care of nephrologists are a prioritized group, often with a known underlying renal diagnosis. They may undergo more frequent blood tests, which may increase the likelihood of finding incidental changes in serum creatinine (i.e. not prompted by clinical condition) or non-meaningful changes in creatinine (false-positive AKI episodes) [7]. 

Clinical evidence is important for guiding clinical decisions both directly (risk prediction tools [8–10]) and indirectly (informing clinical guidelines [2]). Risk tools, such as the kidney failure risk equation (KFRE), can be helpful adjuncts for triaging patients and reducing waiting times [11]. However, clinicians should be mindful that evidence generated from one context (people in an unselected population) may not be generalizable to others (selected people already in nephrology clinics) [12]. It is therefore important to understand the prognostic implications of small changes in creatinine that occur among people under nephrology clinic care and whether these implications vary according to underlying renal diagnosis, age or other parameters. It is also unclear if, in the setting of recent AKI, prognosis can still be adequately described by established decision tools [8–10] or if previous episodes of AKI should be factored in, particularly when deciding if someone with recent AKI but otherwise preserved kidney function should receive ongoing clinic care [13].

The purpose of this analysis was to evaluate the impact of small changes in serum creatinine (AKI episodes according to established definitions) on the long-term outcomes of those already under the care of nephrologists. First, we determined whether AKI was independently associated with kidney failure or death (a competing risk) in this population. Then we evaluated the incremental improvement of adding AKI to established predictors in the KFRE for predicting kidney failure among those already under nephrology care.

## MATERIALS AND METHODS

### Population

The Patient Records and Outcome Management Information System (PROMIS) collects health data from all people with kidney disease who receive care from a nephrologist in a kidney care clinic in any of 41 renal units in British Columbia (population ∼4.5 million) [14]. People receive tests with protocolized regularity with minimum blood test frequencies in accordance with international guidelines [15]. This unique province-wide dataset allows insight into the prognosis of people directly cared for by nephrologists. For these people, the dataset includes blood tests both from clinic visits and hospitalizations. For this analysis, the population comprised people >18 years of age who entered follow-up in PROMIS from 1 January 2003 to 31 July 2009 and who remained alive after a 2-year observation period without need for long-term RRT or estimated glomerular filtration rate (eGFR) <15 mL/min/1.73 m^2^. The study was approved by the Providence Health Care Research Ethics Board.

### Exposure

The exposure was an episode of AKI during 2 years of initial observation from the date of first entry into PROMIS. The comparator was no AKI episode. We used a Kidney Disease: Improving Global Outcomes (KDIGO)-based definition for AKI incorporating a hierarchy of serum creatinine changes. This definition for AKI has previously been described in detail elsewhere [4]. Briefly, the definition involves one of three criteria: (i) serum creatinine ≥1.5 times higher than the median of all creatinine values 8–90 days ago or 91–365 days ago if no tests between 8 and 90 days; (ii) serum creatinine ≥1.5 times higher than the lowest creatinine within 7 days and (iii) serum creatinine >26 µmol/L higher than the lowest creatinine within 48 h.

### Outcomes

Subsequent outcomes were kidney failure and death without kidney failure. Kidney failure was defined as either long-term RRT or an eGFR <15 mL/min/1.73 m^2^ sustained for at least 90 days [15]. For eGFR we used the Chronic Kidney Disease Epidemiology Collaboration (CKD-EPI) creatinine equation [16].

### Follow-up

To avoid immortal time bias, follow-up started at the end of a 2-year observation period (illustrated in Supplementary data, Figure S1). Follow-up continued until the first outcome or for a further 5 years (i.e. up to July 2016).

### Covariates

Covariates were age, sex and eGFR at baseline and after the 2-year observation period, proteinuria, blood pressure (systolic and diastolic), history of diabetes mellitus and primary renal diagnosis as recorded at baseline in the PROMIS database. Proteinuria was determined using urine albumin/creatinine measurements [15].

### Statistical analyses

We reported the number of people who developed AKI by each of the three main definition critera, by AKI severity stage and by the presence of recurrent episodes. We compared the characteristics of people with and without AKI during the 2-year observation period and determined the proportion of people with kidney failure, death without kidney failure (competing risk) or alive without kidney failure after 5 years of follow-up (i.e. 7 years from the date of entry into PROMIS). For clarity of presentation in the characteristics tables, proteinuria was grouped as severe, moderate, none/mild and unrecorded (albumin:creatinine ratio >300, 30–300, <30 mg/g and unrecorded, respectively) and eGFR was grouped with CKD stage groups based on KDIGO CKD criteria [15]. Both covariates were analysed continuously in the prediction models.

For those with and without AKI, we plotted cumulative incidence functions for kidney failure stratified by age, eGFR and underlying renal diagnosis (recognized predictors of kidney failure) [9]. We fitted Cox models for the cause-specific hazard ratio (HR) (AKI versus no AKI) for kidney failure and the competing risk of death without kidney failure adjusted for covariates. Continuous variables were assessed for non-linearity visually and using fractional polynomials [17]. The proportional hazards assumption was verified by −ln{−ln(survival)} plots.

As kidney failure is potentially a rare endpoint, in a sensitivity analysis we used a recognized alternative endpoint: 30% sustained decrease in eGFR or long-term RRT [18]. In addition, as both competing outcomes may vary by AKI phenotype, in subgroup analyses we limited our definition of AKI to the third criterion (small changes of serum creatinine >26 µmol/L in 48 h), to only those with AKI severity stage 1 (stages 2 and 3 excluded) and to only those with recurrent AKI episodes during the 2-year observation period.

For prediction models of 5-year kidney failure risk we externally validated the widely used 5-year KFRE [8, 9] in the nephrology clinic cohort and compared its performance with two refitted models developed in the same cohort. For one refitted model we used the same variables as the four-variable KFRE (age, sex, eGFR and albumin:creatinine ratio) and in the second we added AKI as an additional variable. As death is a competing risk for kidney failure, competing risks regression models were constructed using the Fine and Gray technique [19]. The association of AKI (versus no AKI) with poor outcomes is greater at higher eGFRs [4, 5], and people with an eGFR <30 mL/min/1.73 m^2^ typically receive regular nephrology clinic care (per current guidelines) [15] for complications of CKD even if kidney failure risk is low. Therefore our analyses were stratified by eGFR (</≥30 mL/min/1.73 m^2^) and prediction models assessing incremental improvement in AKI focused on those with eGFR ≥30 mL/min/1.73 m^2^.

To assess model performance we compared discrimination of these models by calculating the area under the receiver operated characteristics curve (AUC). The value of an AUC lies between 0.5 and 1, with 0.5 indicating no better than a coin toss and 1 indicating perfect discrimination between those with and without an outcome [20]. We also evaluated calibration by plotting the mean observed proportion of people with the outcomes against the predicted probabilities of the outcome by increasing tenths of predicted probabilities. Further, for the external validation of KFRE we calculated the calibration slope. This is the coefficient of a regression model containing the linear predictor of the KFRE [21]. A slope close to 1 indicates excellent agreement, whereas a slope <1 may indicate unduly extreme predictions and a slope >1 may indicate predictions that do not vary sufficiently for that model [22, 23].

Even if a model discriminates well overall, this may not lead to better decisions about the appropriate threshold needed for clinical use. For instance, a new model may be useful at a risk threshold cut-off of 80%, but if intervention is recommended when the risk of an outcome is lower (e.g. 10%), the same model may no longer be useful [24]. Decision curve analysis is a method of assessing the clinical usefulness of different models at an appropriate (preferably prespecified) threshold compared with strategies of ‘treat all’ or ‘treat none’ [25]. It is a plot of the ‘net benefit’ of each model, which is a trade-off between true positives and false positives at different thresholds described by the equation below [25]. ‘Treatment’ could be a new drug, a procedure or a decision to follow-up.

$$Net\;benefit=\left(\frac{true\;positive}{total\;sample\;size}\right)-\;\left[\left(\frac{false\;positive}{total\;sample\;size}\right)\;\times \left(\frac{threshold\;probability}{1-threshold\;probability}\right)\right]$$

Elsewhere, a 5-year KFRE risk threshold ≥3% has been suggested and used as a criterion for eligible transition from primary care to nephrology care among people with an eGFR ≥30 mL/min/1.73 m^2^ [11]. Decision curve analysis should therefore show a net benefit at this threshold. Analyses were performed in Stata SE 13 (StataCorp, College Station, TX, USA) [26].

## RESULTS

### Population

**FIGURE 1:**
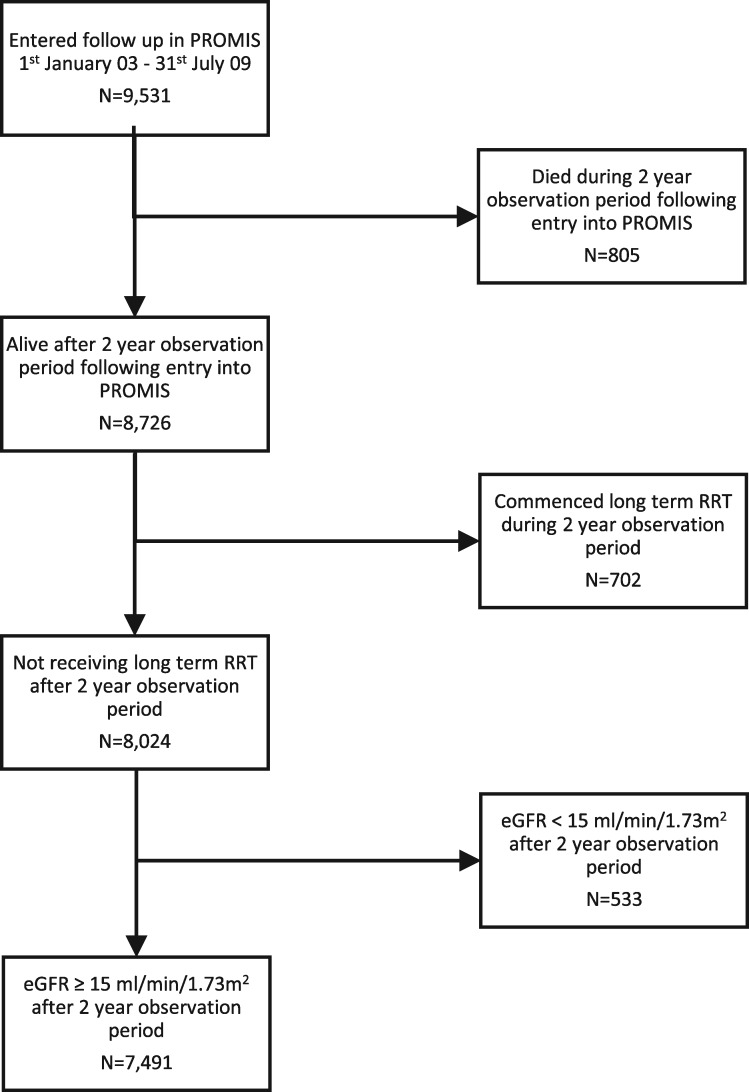
Development of the study population.

From a population of ∼4.5 million, there were 9531 people who were registered into a nephrologist’s care in PROMIS between 1 January 2003 and 31 July 2009, including 8726 who were still alive after 2 years of observation. As shown in Figure 1, after excluding those who were already receiving long-term RRT or had an eGFR <15 mL/min/1.73 m^2^, 7491 people met the study inclusion criteria, including 995 (13.3%) with an episode of AKI. Subsequent follow-up was for a median of 5 years, or 27 742 patient-years. A breakdown of AKI by definition criteria, severity stage and recurrence of AKI episodes is provided in Supplementary data, Table S1.

### Baseline characteristics of people with and without AKI

Table 1 describes the characteristics of people with and without an episode of AKI during the initial 2-year observation period prior to study follow-up. The proportion of people with AKI was no greater among older patients than younger patients and no different for women and men. In this cohort, AKI episodes occurred more frequently among those with baseline eGFR ≥60 versus <30 mL/min/1.73 m^2^ (18.8% versus 13.2%). There were also more AKI episodes among people with severe proteinuria and high blood pressure. Occurrence of AKI varied by underlying renal diagnosis (from 4.4% of people with polycystic kidney disease to 17.0% of people with diabetes-related kidney disease).

**Table 1:** Characteristics of people with and without AKI during the 2-year observation period

	Overall, *N*		AKI in first 2 years		No AKI in first 2 years	
			*N*	%	*N*	%
*N*	7491		995		6496	
Age (years)						
≥80	1488		162	16.3	1326	20.4
60–79	4365		617	62.0	3748	57.7
<60	1638		216	21.7	1422	21.9
Sex						
Female	3451		531	53.4	3509	54.0
Male	4040		464	46.6	2987	46.0
Baseline eGFR (mL/min/1.73 m^2^						
<30	2672		352	35.4	2320	35.7
30–44	2954		367	36.9	2587	39.8
45–59	1142		140	14.1	1002	15.4
≥60	723		136	13.7	587	9.0
Baseline proteinuria						
Severe	1415		229	23.0	1186	18.3
Moderate	1873		271	27.2	1602	24.7
None/mild	1570		207	20.8	1363	21.0
Not tested	2633		288	28.9	2345	36.1
Systolic blood pressure (mmHg						
≥160	911		134	13.5	777	12.0
120–160	3603		487	48.9	3116	48.0
<120	1235		177	17.8	1058	16.3
Not recorded	1742		197	19.8	1545	23.8
Diastolic blood pressure (mmHg						
≥100	148		24	2.4	124	1.9
60–100	5014		687	69.0	4327	66.6
<60	587		87	8.7	500	7.7
Not recorded	1742		197	19.8	1545	23.8
Diabetes mellitus						
Yes	4511		707	71.1	3804	58.6
No	2980		288	28.9	2692	41.4
Primary renal diagnosis						
Diabetes mellitus	1220		207	20.8	1013	15.6
Glomerulonephritis	368		44	4.4	324	5.0
Interstitial/intrinsic renal	699		101	10.2	598	9.2
Multisystem disorder	430		67	6.7	363	5.6
Polycystic kidney disease	159		7	0.7	152	2.3
Vascular/hypertension	1636		225	22.6	1411	21.7
Uncertain aetiology	1059		134	13.5	925	14.2
Not recorded	1920		210	21.1	1710	26.3
Year of first follow-up						
2003–06	3139		440	44.2	2699	41.5
2007–09	4352		555	55.8	3797	58.5

All people in this analysis were observed for 2** **years for the development of AKI. Baseline refers to covariate values at the beginning of a 2-year observation period prior to study follow- up.

### Overall 5-year outcomes among people with and without AKI

Five-year outcomes are reported in Table 2, stratified by age, sex, eGFR and other characteristics. Overall, people with AKI in the preceding 2 years (versus no AKI) had an increased risk of kidney failure (33.1% versus 26.3%) and the competing outcome of death (23.8% versus 16.8%) (χ^2^ P < 0.001; Table 2). This pattern was present irrespective of age, sex, eGFR (as measured after the 2-year observation period), proteinuria and renal diagnosis.

**Table 2:** Subsequent 5-year outcomes among people with and without an episode of AKI in the previous 2-year observation period

		AKI in previous 2 years				No AKI in previous 2 years			
	Overall, *N*	AKI, *N*	Alive at 5 years, %	Developed kidney failure, %	Died without kidney failure, %	No AKI, *N*	Alive at 5 years, %	Developed kidney failure, %	Died without kidney failure, %
*N*	7491	995	43.1	33.1	23.8	6496	56.9	26.3	16.8
Age (years									
≥80	1488	162	35.8	27.2	37.0	1326	41.2	23.0	35.8
60–79	4365	617	41.0	33.4	25.6	3748	59.0	25.6	15.4
<60	1638	216	54.6	36.6	8.8	1422	66.0	30.8	3.2
Sex									
Female	3451	531	49.8	30.0	20.3	3509	59.1	25.4	15.5
Male	4040	464	37.3	35.8	26.9	2987	55.0	27.0	18.0
Post-observation eGFR^a^ (mL/min/1.73 m^2^)									
<30	3015	473	25.6	51.2	23.2	2542	31.9	47.7	20.4
30–44	2812	357	55.2	18.2	26.6	2455	66.3	15.4	18.2
45–59	1057	109	68.8	12.8	18.3	948	81.4	7.9	10.7
≥60	607	56	64.3	14.3	21.4	551	88.2	7.1	4.7
Post-observation proteinuria^a^									
Severe	1468	233	18.0	62.7	19.3	1235	24.3	57.1	18.6
Moderate	1824	277	34.2	29.6	36.1	1547	44.7	25.1	30.3
None/mild	1566	197	41.1	22.3	36.5	1369	63.7	12.9	23.4
Not tested	2633	288	73.3	19.8	6.9	2345	78.2	18.6	3.2
Systolic blood pressure (mmHg)									
≥160	911	134	35.8	41.8	22.4	777	48.3	36.8	14.9
120–160	3603	487	40.2	41.7	18.1	3116	53.7	31.6	14.7
<120	1235	177	53.1	20.9	26.0	1058	58.4	23.9	17.7
Not recorded	1742	197	46.2	16.8	37.1	1545	66.7	11.8	21.6
Diastolic blood pressure (mmHg)									
≥100	148	24	37.5	54.2	8.3	124	58.1	32.3	9.7
60–100	5014	687	43.7	36.7	19.7	4327	54.7	30.6	14.7
<60	587	87	33.3	35.6	31.0	500	45.4	31.6	23.0
Not recorded	1742	197	46.2	16.8	37.1	1545	66.7	11.8	21.6
Diabetes mellitus									
Yes	4511	707	40.7	35.4	23.9	3804	52.7	30.8	16.5
No	2980	288	48.9	27.4	23.6	2692	62.8	19.9	17.3
Primary renal diagnosis									
Diabetes mellitus	1220	207	34.8	50.2	15.0	1013	45.3	43.4	11.3
Glomerulonephritis	368	44	25.0	61.4	13.6	324	50.3	46.0	3.7
Interstitial/intrinsic renal	699	101	53.5	29.7	16.8	598	60.9	28.8	10.4
Multisystem disorder	430	67	37.3	38.8	23.9	363	59.2	20.4	20.4
Polycystic kidney disease	159	7	42.9	57.1	0.0	152	46.7	52.6	0.7
Vascular/hypertension	1636	225	49.3	25.3	25.3	1411	57.3	27.6	15.1
Uncertain aetiology	1059	134	44.0	29.1	26.9	925	63.3	20.5	16.1
Not recorded	1920	210	44.8	20.0	35.2	1710	60.2	12.3	27.4
Year of first follow-up									
2003–06	3139	440	44.5	32.3	23.1	2699	58.7	25.4	15.9
2007–09	4352	555	42.0	33.7	24.3	3797	55.6	26.9	17.5

All people in this analysis were observed for 2** **years for the development of AKI. Outcomes refer to CKD stage 5 and death over the subsequent 5** **years thereafter.

a Refers to the last measurement from the 2-year observation period.

### Cumulative rates of kidney failure and the competing event of death

**FIGURE 2:**
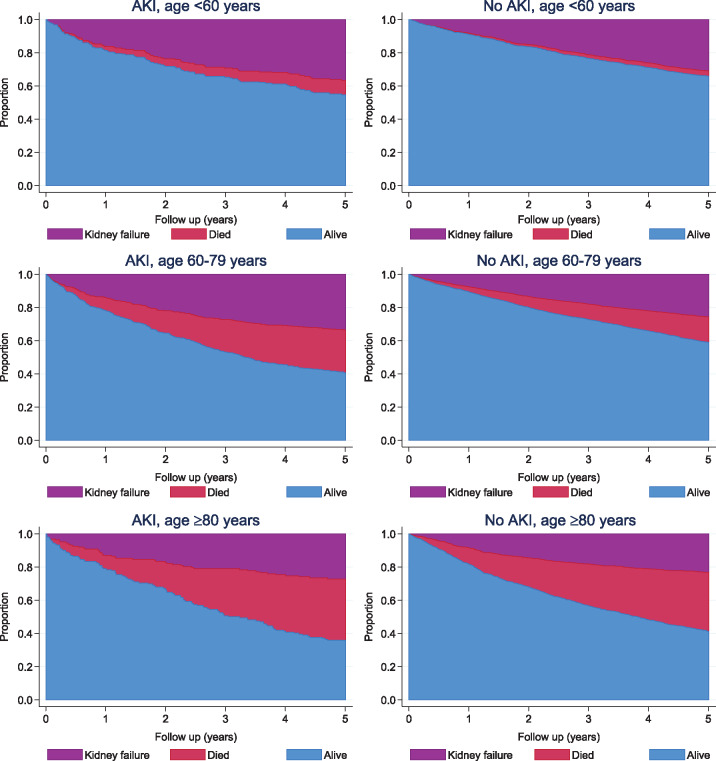
Cumulative incidence of kidney failure and death among those with and without AKI grouped by age (years).

Figure 2 and Supplementary data, Figures S2 and S3 show the cumulative incidences of kidney failure and death without kidney failure in those with and without AKI, stratified by age, eGFR and primary renal diagnosis, respectively. Stratification by age showed an increase in both kidney failure and death after AKI in all age groups (Figure 2). Those ≥80 years of age were more likely to die than reach kidney failure, whereas death was uncommon among those <60 years of age. AKI was also associated with poorer outcomes irrespective of eGFR (Supplementary data, Figure S2) and renal diagnosis (Supplementary data, Figure S3), but the occurrence of the competing event of death varied between subgroups.

### Independent association between AKI and kidney failure and death

Table 3 describes the independent role of AKI (versus no AKI) in kidney failure and the competing event of death with stepwise adjustment for age, sex, eGFR, proteinuria and primary renal diagnosis. As eGFR has previously been shown to modify the prognosis of AKI [4, 5, 27], two separate models were built for those with eGFR ≥30 mL/min/1.73 m^2^ and those with eGFR <30 mL/min/1.73 m^2^. Irrespective of eGFR, AKI was associated with kidney failure in univariable analysis, however, in multivariable analysis the excess risk associated with AKI among those with eGFR <30 mL/min/1.73 m^2^ could be explained by adjusting for covariates [HR 1.05 (95% CI 0.91–1.21)]. By comparison, among those with eGFR ≥30 mL/min/1.73 m^2^, even after adjusting for potential confounders, those with previous AKI remained at an excess risk [HR 1.35 (95% CI 1.07–1.70)]. In a sensitivity analysis using a recognized alternative metric of CKD progression (30% eGFR decline or long-term RRT), there was a similar pattern of excess risk for those with AKI and eGFR ≥30 mL/min/1.73 m^2^ (Supplementary data, Table S2). In subgroup analyses (Supplementary data, Tables S3–S5) we found a similar pattern of a large excess risk of death and small excess risk of kidney failure even when our definition of AKI was restricted to a criterion involving small creatinine changes (>26 µmol/L) within 48 h. Among those with eGFR ≥30 mL/min/1.73 m^2^, the excess kidney failure risk was substantial for the minority of people who had recurrent AKI episodes [adjusted HR 2.34 (95% CI 1.50–3.66); Supplementary data, Table S5].

**Table 3:** Independent role of previous AKI in subsequent kidney failure and competing risk of death

Comparison	Subgroup	Adjusted covariates	AKI versus no AKI for kidney failure, HR (95% CI)	AKI versus no AKI for death without kidney failure, HR (95% CI)
AKI versus no AKI	eGFR < 30	–	1.24 (1.08–1.42)	1.34 (1.09–1.65)
AKI versus no AKI	eGFR < 30	Age and sex	1.23 (1.07–1.42)	1.46 (1.19–1.79)
AKI versus no AKI	eGFR < 30	Age, sex and eGFR	1.10 (0.95–1.26)	1.43 (1.17–1.76)
AKI versus no AKI	eGFR < 30	Age, sex, eGFR and proteinuria	1.03 (0.89–1.18)	1.43 (1.16–1.76)
AKI versus no AKI	eGFR < 30	Age, sex, eGFR, proteinuria and renal diagnosis	1.05 (0.91–1.21)	1.43 (1.16–1.75)
AKI versus no AKI	eGFR ≥30	–	1.55 (1.23–1.94)	1.90 (1.57–2.30)
AKI versus no AKI	eGFR ≥30	Age and sex	1.55 (1.24–1.95)	2.10 (1.73–2.54)
AKI versus no AKI	eGFR ≥30	Age, sex and eGFR	1.43 (1.13–1.79)	2.05 (1.69–2.49)
AKI versus no AKI	eGFR ≥30	Age, sex, eGFR and proteinuria	1.34 (1.07–1.69)	1.87 (1.54–2.27)
AKI versus no AKI	eGFR ≥30	Age, sex, eGFR, proteinuria and renal diagnosis	1.35 (1.07–1.70)	1.89 (1.56–2.30)

Age as a linear term, eGFR^-2^, proteinuria in four KDIGO albuminuria categories of severe, moderate, normal/mild and not tested. AKI represents AKI occurring in a 2-year observation period prior to study follow-up. eGFR and proteinuria status were determined from the last available sample at the end of the 2-year observation period. The outcome of kidney failure represents a composite of long-term RRT or eGFR  <15 mL/min/1.73 m^2^ sustained for at least 90 days. eGFR given in mL/min/1.73 m^2^.

### 5 -Year KFRE and refitted models with and without AKI added

**FIGURE 3:**
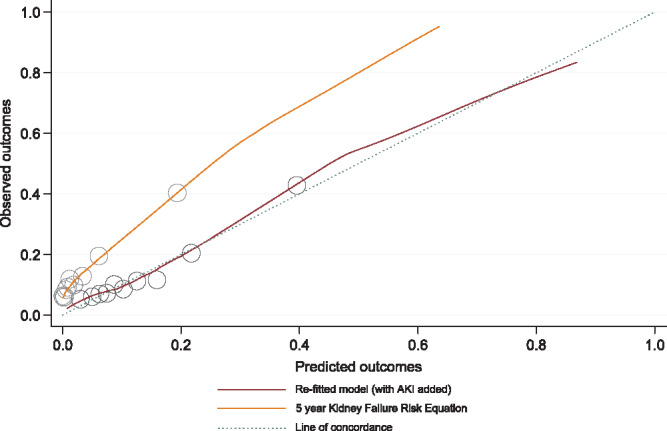
Calibration plots for the 5-year kidney failure risk equation and a refitted model with AKI as an added predictor. Circles represent the cohort grouped in increasing tenths of predicted probabilities. KFRE underpredicted 5-year kidney failure in this cohort.

**FIGURE 4:**
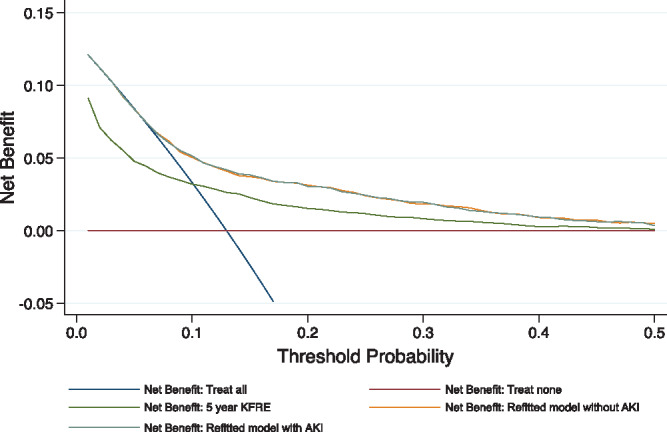
Decision curve analysis of 5-year kidney failure risk prediction models for people with eGFR ≥30 mL/min/1.73 m^2^. KFRE was inferior to a ‘treat all’ approach when the risk threshold was <10%. AKI added no value as an additional predictor in a refitted model.

Table 4 compares three models for predicting kidney failure among people with eGFR ≥30 mL/min/1.73 m^2^: external validation of the 5-year KFRE, a refitted model using the same variables (age, sex, eGFR and proteinuria) developed using the Fine and Gray technique [19] and a second refitted model incorporating AKI as an additional predictor. As shown by the calibration plot (Figure 3) and the calibration slope significantly different from 1 (Table 4), the KFRE was miscalibrated and underpredicted kidney failure in this cohort. For discrimination, respective AUCs were 0.701, 0.715 and 0.716. Using decision curve analysis, the refitted model with AKI was no better than the refitted model without AKI (Figure 4). KFRE was inferior to either refitted model or a ‘treat all’ approach (i.e. clinical follow-up of all people) if used at any risk threshold between 0% and 10%, as suggested in previous studies [11]. Fitting a separate model that included recurrent AKI as a predictor also did not greatly alter discrimination (AUC 0.718).

**Table 4:** Five-year kidney failure risk prediction models for people with eGFR ≥30 mL/min/1.73 m^2^

Model	Equation	Calibration slope^a^	AUC	P-value for AUC comparison with next most complex model
Five-year KFRE	1 −0.924^exp((−0.2201 * (((age)/10) −7.036)) + (0.2467 * (sex−0.5642)) − (0.5567 * ((eGFR/5) −7.222)) + ((0.451 * (lnACR−5.137))))	0.44 (0.39–0.49)	0.701 (0.676–0.725)	0.02
Refitted model without AKI	1 −0.916^exp((−0.3074 * ((age−70)/10)) + (−0.1801 * ((eGFR−30)/5)) + (0.2227 * sex) + (0.2292 * lnACR))	1.00 (0.90–1.10)	0.715 (0.691–0.739)	0.16
Refitted model with AKI	1 -0.918^exp((−0.307 * ((age−70)/10)) + (−0.179 * ((eGFR−30)/5)) + (0.2224 * sex) + (0.228 * lnACR) + (0.148 * AKI2yr))	1.00 (0.90–1.10)	0.716 (0.692–0.741)	

AKI2 yr represents AKI occurring in a 2-year observation period prior to study follow-up (1 = AKI, 0 = no AKI). Age (in years), eGFR and proteinuria were determined from the last available sample at the end of the 2-year observation period. Sex = 1 if male and 0 if female. The outcome of kidney failure represents a composite of long-term RRT or eGFR <15 mL/min/1.73 m^2^ sustained for at least 90 days.

a Calibration slope should be expected to be equal to 1 in the development of refitted models (apparent calibration). For external validation of the KFRE, a slope different from 1 indicates miscalibration.

lnACR, natural logarithm of albumin:creatinine ratio (mg/g).

## DISCUSSION

This large long-term analysis of a provincial Canadian cohort is the first to predict kidney failure risk after AKI among people already undergoing nephrology clinic follow-up. There are two important messages for nephrologists.

First, small acute creatinine changes of AKI in people already under nephrology care are associated with a small independent increased risk of kidney failure and a large competing risk of death, irrespective of age or renal diagnosis. Incorporating AKI into kidney failure risk prediction models does not improve predictions, but the reason is likely to be because many people with AKI die before kidney failure. Thus, while AKI may not substantially alter kidney failure risk predictions, the use of prediction models in individuals without an appreciation of the full impact of AKI would be simplistic. Thus the notion of ‘competing risk of death’ needs to be incorporated more overtly into clinical thinking and discussions with patients.

Second, the established KFRE calibrated poorly when used in people with preserved eGFR (≥30 mL/min/1.73 m^2^) under nephrology clinic follow-up, leading to substantially underpredicted risks. A strategy of caring for all would be better than using the KFRE at a threshold of 3% to prioritize people with eGFR ≥30 mL/min/1.73 m^2^ who should continue to receive nephrology care. Reasons for this are unclear, but people with eGFR ≥30 mL/min/1.73 m^2^ who are followed by nephrologists may already be a high-risk group for reasons not completely captured by the KFRE. In keeping with this, AKI did not vary with age in this cohort, even though old age is an established risk factor for AKI (and death). This suggests selective nephrology follow-up of those in the general population who were most likely to develop kidney failure (rather than die). This complexity indicates that caution is needed when nephrologist apply prediction tools to individual people in specific clinical contexts.

This analysis shows that clinicians should understand better the substantial competing risk of death occurring after AKI before kidney failure can develop. That is, AKI may serve as a ‘signal’ for adverse outcomes, including but not limited to kidney failure. As illustrated in the cumulative incidence plots of this study, this increased mortality is present irrespective of age, eGFR or underlying renal diagnosis. The substantial competing outcomes after AKI should prompt us to consider the numerous reasons, other than simply kidney failure risk, that people with kidney disease may benefit from nephrology care. Nephrology care may include prevention of recurrent acute illness episodes, prevention of CKD complications, optimization of cardiovascular profile, fluid balance and alleviation of symptoms. To date, there remains little evidence describing the value of nephrology care in optimizing cardiovascular risk for those with advanced CKD or preventing AKI due to drugs or contrast. Future work should examine this for the high-risk group identified in this analysis of AKI survivors.

Our analysis also highlights the importance of clinical context when evaluating AKI. Even if there is a similar pattern of increased risk after AKI across numerous studies, absolute risk differs depending on the clinical context. For instance, the AKI definition in this analysis (in a nephrology clinic population) was identical to that used in previous work (in a general population), but in this analysis the risk of renal progression was substantially higher and the risk of mortality was lower [5].

Strengths of this analysis include the large sample size, covering an entire province in Canada. The novel focus on those under nephrology care makes the findings particularly relevant for practising nephrologists and multidisciplinary kidney care clinics. The definition of AKI was comprehensive and KDIGO based and modelling included measures of proteinuria that are frequently unavailable in clinical AKI studies [3, 6, 28]. Moreover, an opportunity for eGFR to return to baseline after AKI (necessary to avoid confusing non-recovery with progression) [5] was provided by using post-AKI episode eGFR values in the analysis after the 2-year observation period. The analysis also can be supported by recent population studies showing the effect modification of AKI outcomes by eGFR in the USA and UK [4, 27] and recent work illustrating the potential value of updating risk prediction tools as new information arises [29].

The analysis also has limitations. This analysis involves AKI occurring while under nephrology care and does not include episodes of AKI occurring prior to nephrology referral (which may have prompted referral). This may have led to an attenuation of the association between AKI and outcomes of interest. We recognize that clinical nephrology practices in British Columbia may vary from practices in other countries, although our findings are broadly consistent with a methodologically similar study using a UK cohort [5]. We also recognize that as the covariates were obtained from routinely collected health data, not all items were complete (e.g. proteinuria). This, however, reflects real-world clinical practice in nephrology clinics in British Columbia, where not all urine samples are sent for quantification, e.g. when bedside urinalysis is normal. This explains why those without a documented measure of proteinuria had the best outcomes. Additional adjustment for a wider range of comorbidities was also not possible, but we note that wider comorbidities are not a component of the KFRE either. Finally, we note that we tested our prediction models in just one cohort, but this is sufficient for the purpose of our study: for evaluating incremental risk with AKI as a predictor, we used the same dataset and variables for two refitted models so that the only difference between the models was the inclusion of the predictor AKI. For evaluating the use of KFRE, we externally validated an equation already studied in numerous population datasets.

In conclusion, a history of AKI based on small creatinine changes (versus no AKI) carries a poorer long-term prognosis for all outcomes among those already under nephrology clinic care, irrespective of the level of kidney function, age and renal diagnosis. People with CKD and AKI have risks beyond simply kidney failure, including cardiovascular events and death. Clinical appreciation of these other risks and targeted research to identify the best strategies to modify risks in high-risk individulas is critical.

## SUPPLEMENTARY DATA

Supplementary data are available at ndt online.

## Supplementary Material

gfy294_Supplementary_Data

## References

[1] Chertow GM , Burdick E, Honour M et al Acute kidney injury, mortality, length of stay, and costs in hospitalized patients. J Am Soc Nephrol 2005; 16: 3365–3370 16177006 10.1681/ASN.2004090740

[2] Kidney Disease: Improving Global Outcomes (KDIGO) Acute Kidney Injury Work Group. KDIGO clinical practice guideline for acute kidney injury. Kidney Int Suppl 2012; 2: 1–138

[3] Coca SG , Singanamala S, Parikh CR. Chronic kidney disease after acute kidney injury: a systematic review and meta-analysis. Kidney Int 2012; 81: 442–448 22113526 10.1038/ki.2011.379 PMC3788581

[4] Sawhney S , Marks A, Fluck N et al Intermediate and long-term outcomes of survivors of acute kidney injury episodes: a large population-based cohort study. Am J Kidney Dis 2017; 69: 18–28 27555107 10.1053/j.ajkd.2016.05.018 PMC5176133

[5] Sawhney S , Marks A, Fluck N et al Post-discharge kidney function is associated with subsequent ten-year renal progression risk among survivors of acute kidney injury. Kidney Int 2017; 92: 440–452 28416224 10.1016/j.kint.2017.02.019 PMC5524434

[6] Sawhney S , Mitchell M, Marks A et al Long-term prognosis after acute kidney injury (AKI): what is the role of baseline kidney function and recovery? A systematic review. BMJ Open 2015; 5: e006497 10.1136/bmjopen-2014-006497 PMC4289733 25564144

[7] Lin J , Fernandez H, Shashaty MGS et al False-positive rate of AKI using consensus creatinine-based criteria. Clin J Am Soc Nephrol 2015; 10: 1723–1731 26336912 10.2215/CJN.02430315 PMC4594067

[8] Tangri N , Stevens LA, Griffith J et al A predictive model for progression of chronic kidney disease to kidney failure. JAMA 2011; 305: 1553–1559 21482743 10.1001/jama.2011.451

[9] Tangri N , Grams ME, Levey AS et al Multinational assessment of accuracy of equations for predicting risk of kidney failure: a meta-analysis. JAMA 2016; 315: 164–174 26757465 10.1001/jama.2015.18202 PMC4752167

[10] Tangri N , Kitsios GD, Inker LA et al Risk prediction models for patients with chronic kidney disease. A systematic review. Ann Intern Med 2013; 158: 596–603 23588748 10.7326/0003-4819-158-8-201304160-00004

[11] Tangri N , Ferguson T, Komenda P. Pro: Risk scores for chronic kidney disease progression are robust, powerful and ready for implementation. Nephrol Dial Transplant 2017; 32: 748–751 28499025 10.1093/ndt/gfx067

[12] O’Hare AM , Hotchkiss JR, Kurella Tamura M et al Interpreting treatment effects from clinical trials in the context of real-world risk information: the example of end-stage renal disease prevention in older adults. JAMA Intern Med 2014; 174: 391–397 24424348 10.1001/jamainternmed.2013.13328 PMC4119007

[13] Silver SA , Adu D, Agarwal S et al Strategies to enhance rehabilitation after acute kidney injury in the developing world. Kidney Int Rep 2017; 2: 579–593

[14] Lafrance JP , Djurdjev O, Levin A. Incidence and outcomes of acute kidney injury in a referred chronic kidney disease cohort. Nephrol Dial Transplant 2010; 25: 2203–2209 20124548 10.1093/ndt/gfq011

[15] Kidney Disease: Improving Global Outcomes CKD Work Group. KDIGO clinical practice guideline for the evaluation and management of chronic kidney disease. Kidney Int Suppl 2013; 3: 1–150

[16] Levey AS , Stevens LA, Schmid CH et al A new equation to estimate glomerular filtration rate. Ann Intern Med 2009; 150: 604–612 19414839 10.7326/0003-4819-150-9-200905050-00006 PMC2763564

[17] Sauerbrei W , Royston P, Binder H. Selection of important variables and determination of functional form for continuous predictors in multivariable model building. Stat Med 2007; 26: 5512–5528 18058845 10.1002/sim.3148

[18] Grams ME , Sang Y, Coresh J et al Candidate surrogate end points for ESRD after AKI. J Am Soc Nephrol 2016; 27: 2851–2859 26857682 10.1681/ASN.2015070829 PMC5004655

[19] Fine JP , Gray RJ. A proportional hazards model for the subdistribution of a competing risk. J Am Stat Assoc 1999; 94: 496–509

[20] Austin PC , Steyerberg EW. Interpreting the concordance statistic of a logistic regression model: relation to the variance and odds ratio of a continuous explanatory variable. BMC Med Res Methodol 2012; 12: 82 22716998 10.1186/1471-2288-12-82 PMC3528632

[21] van Houwelingen HC. Validation, calibration, revision and combination of prognostic survival models. Stat Med 2000; 19: 3401–3415 11122504 10.1002/1097-0258(20001230)19:24<3401::aid-sim554>3.0.co;2-2

[22] Steyerberg EW. Clinical Prediction Models. 1st edn New York: Springer, 2009

[23] Debray TPA , Verguwe Y, Koffijberg H et al A new framework to enhance the interpretation of external validation studies of clinical prediction models. J Clin Epidemiol 2015; 68: 279–289 25179855 10.1016/j.jclinepi.2014.06.018

[24] Steyerberg EW , Vergouwe Y. Towards better clinical prediction models: seven steps for development and an ABCD for validation. Eur Heart J 2014; 35: 1925–1931 24898551 10.1093/eurheartj/ehu207 PMC4155437

[25] Vickers AJ , Van Calster B, Steyerberg EW. Net benefit approaches to the evaluation of prediction models, molecular markers, and diagnostic tests. Br Med J 2016; 352 10.1136/bmj.i6 PMC4724785 26810254

[26] StataCorp. Stata Statistical Software: Release 13. College Station, TX: StataCorp, 2013

[27] Lafrance JP , Miller DR. Acute kidney injury associates with increased long-term mortality. J Am Soc Nephrol 2010; 21: 345–352 20019168 10.1681/ASN.2009060636 PMC2834549

[28] Coca SG , Yusuf B, Shlipak MG et al Long-term risk of mortality and other adverse outcomes after acute kidney injury: a systematic review and meta-analysis. Am J Kidney Dis 2009; 53: 961–973 19346042 10.1053/j.ajkd.2008.11.034 PMC2726041

[29] Tangri N , Inker LA, Hiebert B et al A dynamic predictive model for progression of CKD. Am J Kidney Dis 2017; 69: 514–520 27693260 10.1053/j.ajkd.2016.07.030

