# Supplementary material for: Predicting kidney failure risk after acute kidney injury among people receiving nephrology clinic care
Source: Nephrol Dial Transplant. 2018 Oct 15;35(5):836–45. doi: 10.1093/ndt/gfy294 (PMC7203563; doi:10.1093/ndt/gfy294)
Supplement: gfy294_Supplementary_Data [file gfy294_supplementary_data.docx]

# Predicting kidney failure risk after AKI among people receiving nephrology clinic care

Supplementary material

# Supplementary material

Supplementary figure 1 –Time periods for AKI exposure status and subsequent outcomes


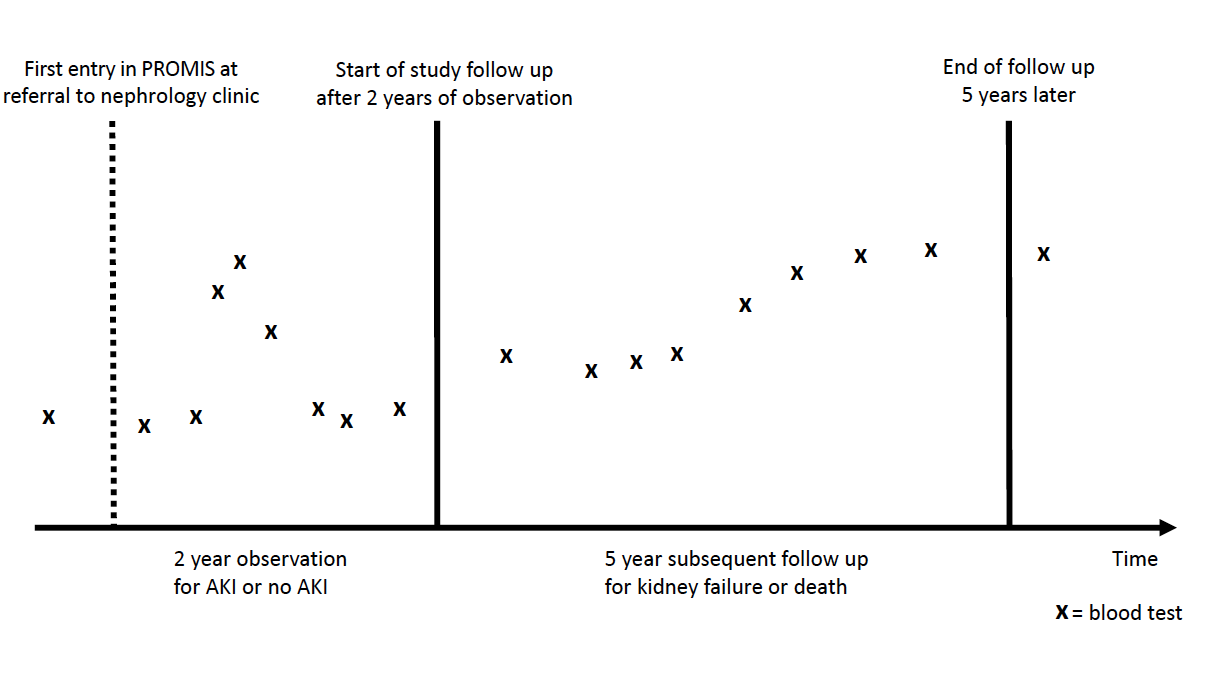


Supplementary figure 2 – Cumulative incidence of kidney failure and death among those with and without AKI grouped by baseline eGFR (ml/min/1.73m^2^)


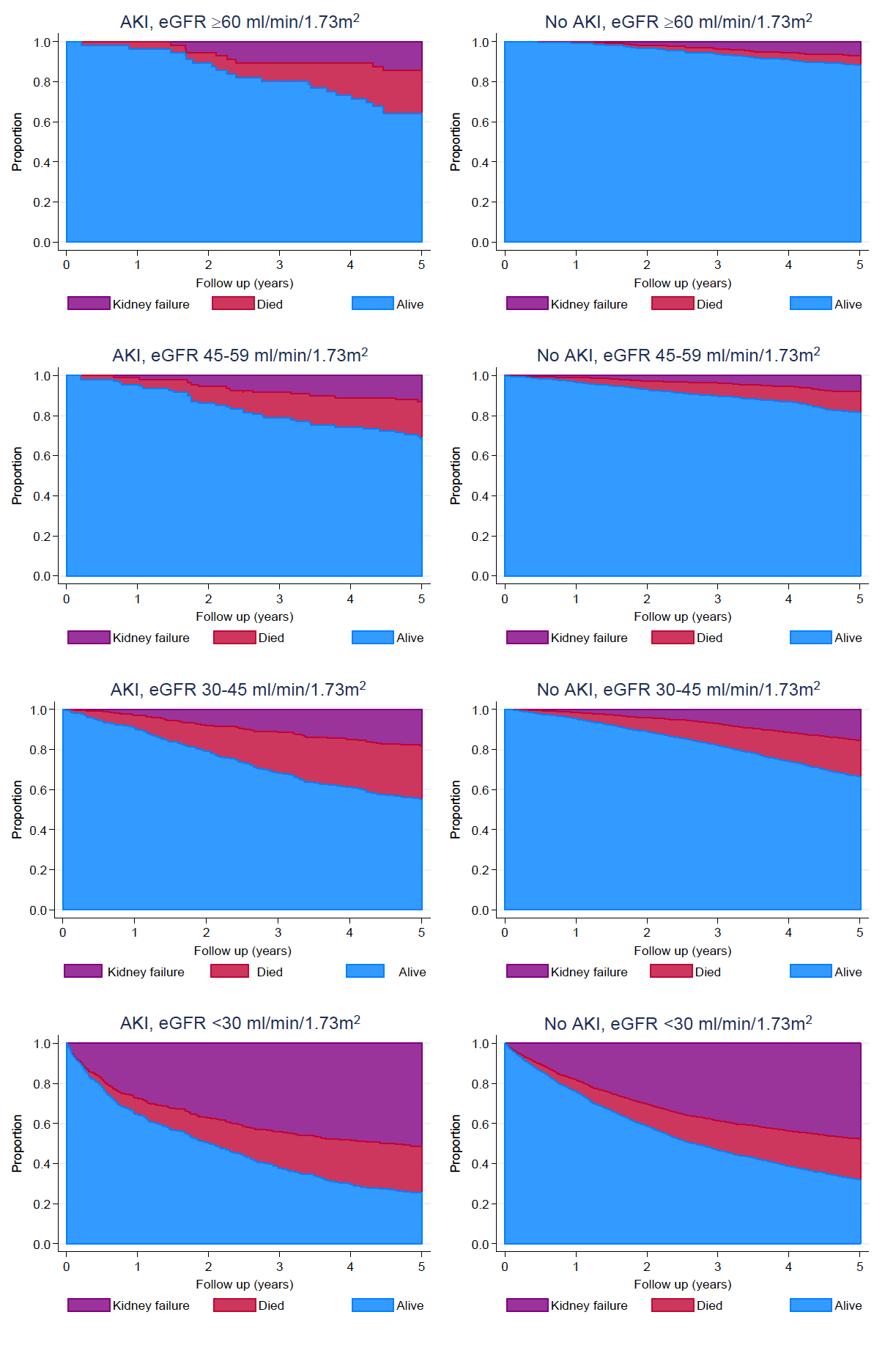


Supplementary figure 3 – Cumulative incidence of kidney failure and death among those with and without AKI grouped by primary renal diagnosis


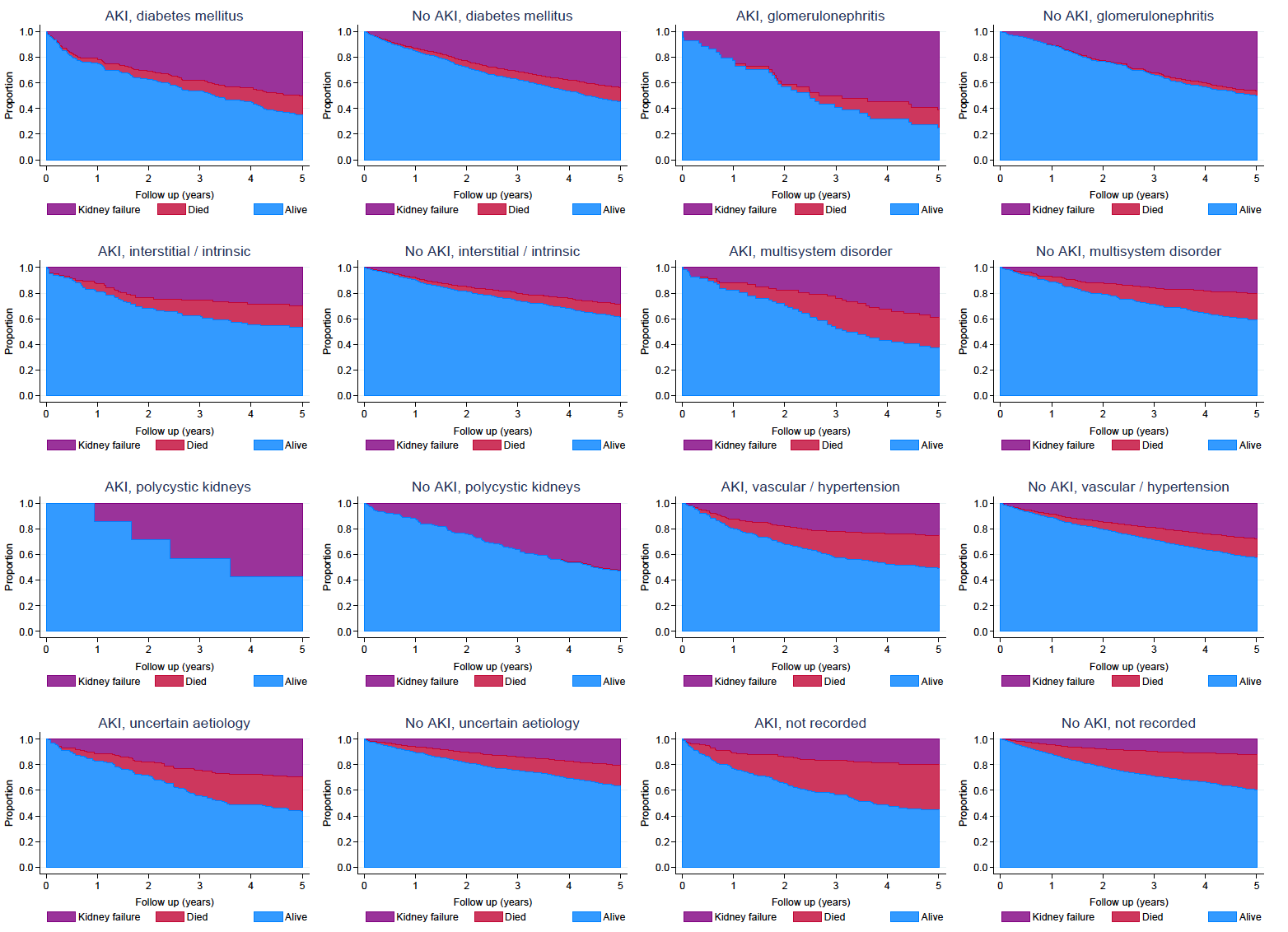


Supplementary table 1 – AKI by definition criteria, severity stage, and recurrence of AKI episode

|  | **AKI in first 2 years** | |
| --- | --- | --- |
|  | **N** | **(%)** |
| **N** | 995 |  |
|  |  |  |
| **AKI criterion** |  |  |
| Scr ≥1.5 times higher than median 8-90 days ago,  or 91-365 days ago if no recent tests | 793 | (79.7) |
| Scr ≥1.5 times higher than the lowest within 7 days | 232 | (23.3) |
| Scr>26 µmol/L higher than the lowest within 48 hours | 422 | (42.4) |
|  |  |  |
| **AKI severity** |  |  |
| Stage 1 | 682 | (68.5) |
| Stage 2 | 146 | (14.7) |
| Stage 3 | 167 | (16.8) |
| **AKI recurrence** |  |  |
| Non-recurrent | 837 | (84.1) |
| Recurrent | 158 | (15.9) |
| *Note*: All people in this analysis were observed for two years for the development of AKI.  AKI criteria are not mutually exclusive  Abbreviations: AKI, acute kidney injury; Scr, serum creatinine | | |

Supplementary table 2 - Sensitivity analysis examining independent role of previous AKI in subsequent 30% eGFR decline (or long term renal replacement therapy) and competing risk of death

| **Comparison** | **Subgroup** | **Adjusted covariates** | **HR AKI vs no AKI   for 30% eGFR decline (95% CI)** | | **HR AKI vs no AKI   for death without 30% eGFR decline (95% CI)** | |
| --- | --- | --- | --- | --- | --- | --- |
| AKI vs no AKI | eGFR<30 | - | 1.13 | (0.96-1.34) | 1.46 | (1.23-1.74) |
| AKI vs no AKI | eGFR<30 | age, sex | 1.10 | (0.93-1.30) | 1.62 | (1.36-1.93) |
| AKI vs no AKI | eGFR<30 | age, sex, eGFR | 1.03 | (0.87-1.21) | 1.59 | (1.33-1.89) |
| AKI vs no AKI | eGFR<30 | age, sex, eGFR, proteinuria | 0.98 | (0.83-1.20) | 1.57 | (1.31-1.87) |
| AKI vs no AKI | eGFR<30 | age, sex, eGFR, proteinuria, renal diagnosis | 1.02 | (0.86-1.21) | 1.56 | (1.31-1.86) |
|  |  |  |  |  |  |  |
| AKI vs no AKI | eGFR≥30 | - | 1.59 | (1.26-2.00) | 1.86 | (1.53-2.25) |
| AKI vs no AKI | eGFR≥30 | age, sex | 1.61 | (1.28-2.05) | 2.08 | (1.72-2.52) |
| AKI vs no AKI | eGFR≥30 | age, sex, eGFR | 1.51 | (1.20-1.91) | 2.05 | (1.69-2.48) |
| AKI vs no AKI | eGFR≥30 | age, sex, eGFR, proteinuria | 1.43 | (1.13-1.81) | 1.83 | (1.51-2.22) |
| AKI vs no AKI | eGFR≥30 | age, sex, eGFR, proteinuria, renal diagnosis | 1.43 | (1.13-1.81) | 1.88 | (1.54-2.28) |
| *Note*: Age as linear term, eGFR^-2^, proteinuria in four KDIGO albuminuria categories of “severe”, “moderate”, “normal/mild” and “not tested”. AKI represents AKI occurring in a 2 year observation period prior to study follow up. eGFR and proteinuria status were determined on the last available sample at the end of the 2 year observation period.  Abbreviations: AKI, acute kidney injury; CI, confidence interval; eGFR, estimated glomerular filtration rate; HR, hazard ratio | | | | | | |

Supplementary table 3 - Subgroup analysis examining independent role of previous AKI when limited only to AKI defined by criterion 3 (>26 µmol/L change in creatinine within 48 hours)

| **Comparison** | **Subgroup** | **Adjusted covariates** | **HR AKI vs no AKI   for kidney failure (95% CI)** | | **HR AKI vs no AKI   for death without kidney failure (95% CI)** | |
| --- | --- | --- | --- | --- | --- | --- |
| AKI vs no AKI | eGFR<30 | - | 1.40 | (1.16-1.70) | 1.49 | (1.12-1.98) |
| AKI vs no AKI | eGFR<30 | age, sex | 1.40 | (1.15-1.69) | 1.53 | (1.15-2.03) |
| AKI vs no AKI | eGFR<30 | age, sex, eGFR | 1.29 | (1.07-1.56) | 1.49 | (1.12-1.99) |
| AKI vs no AKI | eGFR<30 | age, sex, eGFR, proteinuria | 1.22 | (1.01-1.48) | 1.36 | (1.02-1.81) |
| AKI vs no AKI | eGFR<30 | age, sex, eGFR, proteinuria, renal diagnosis | 1.23 | (1.01-1.49) | 1.40 | (1.05-1.87) |
|  |  |  |  |  |  |  |
| AKI vs no AKI | eGFR≥30 | - | 1.73 | (1.28-2.47) | 2.31 | (1.77-3.00) |
| AKI vs no AKI | eGFR≥30 | age, sex | 1.72 | (1.24-2.39) | 2.50 | (1.92-3.25) |
| AKI vs no AKI | eGFR≥30 | age, sex, eGFR | 1.60 | (1.15-2.22) | 2.46 | (1.89-3.21) |
| AKI vs no AKI | eGFR≥30 | age, sex, eGFR, proteinuria | 1.41 | (1.02-1.97) | 2.21 | (1.70-2.88) |
| AKI vs no AKI | eGFR≥30 | age, sex, eGFR, proteinuria, renal diagnosis | 1.48 | (1.07-2.07) | 2.28 | (1.79-2.98) |
| *Note*: Age as linear term, eGFR^-2^, proteinuria in four KDIGO albuminuria categories of “severe”, “moderate”, “normal/mild” and “not tested”. AKI represents AKI occurring in a 2 year observation period prior to study follow up. eGFR and proteinuria status were determined on the last available sample at the end of the 2 year observation period.  Abbreviations: AKI, acute kidney injury; CI, confidence interval; eGFR, estimated glomerular filtration rate; HR, hazard ratio | | | | | | |

Supplementary table 4 - Subgroup analysis examining independent role of previous AKI when limited only to AKI stage 1

| **Comparison** | **Subgroup** | **Adjusted covariates** | **HR AKI vs no AKI   for kidney failure (95% CI)** | | **HR AKI vs no AKI   for death without kidney failure (95% CI)** | |
| --- | --- | --- | --- | --- | --- | --- |
| AKI 1 vs no AKI | eGFR<30 | - | 1.16 | (0.99-1.37) | 1.45 | (1.15-1.84) |
| AKI 1 vs no AKI | eGFR<30 | age, sex | 1.19 | (1.01-1.40) | 1.42 | (1.12-1.79) |
| AKI 1 vs no AKI | eGFR<30 | age, sex, eGFR | 1.12 | (0.95-1.32) | 1.39 | (1.10-1.76) |
| AKI 1 vs no AKI | eGFR<30 | age, sex, eGFR, proteinuria | 1.12 | (0.95-1.32) | 1.41 | (1.12-1.79) |
| AKI 1 vs no AKI | eGFR<30 | age, sex, eGFR, proteinuria, renal diagnosis | 1.11 | (0.94-1.31) | 1.38 | (1.09-1.75) |
|  |  |  |  |  |  |  |
| AKI 1 vs no AKI | eGFR≥30 | - | 1.13 | (0.84-1.51) | 1.86 | (1.49-2.33) |
| AKI 1 vs no AKI | eGFR≥30 | age, sex | 1.15 | (0.86-1.54) | 1.81 | (1.45-2.26) |
| AKI 1 vs no AKI | eGFR≥30 | age, sex, eGFR | 1.19 | (0.89-1.59) | 1.96 | (1.56-2.45) |
| AKI 1 vs no AKI | eGFR≥30 | age, sex, eGFR, proteinuria | 1.26 | (0.94-1.69) | 1.98 | (1.58-2.47) |
| AKI 1 vs no AKI | eGFR≥30 | age, sex, eGFR, proteinuria, renal diagnosis | 1.26 | (0.94-1.68) | 1.86 | (1.49-2.33) |
| *Note*: Age as linear term, eGFR^-2^, proteinuria in four KDIGO albuminuria categories of “severe”, “moderate”, “normal/mild” and “not tested”. AKI represents AKI occurring in a 2 year observation period prior to study follow up. eGFR and proteinuria status were determined on the last available sample at the end of the 2 year observation period.  Abbreviations: AKI, acute kidney injury; CI, confidence interval; eGFR, estimated glomerular filtration rate; HR, hazard ratio | | | | | | |

Supplementary table 5 - Subgroup analysis examining independent role of previous AKI when limited only to recurrent AKI

| **Comparison** | **Subgroup** | **Adjusted covariates** | **HR AKI vs no AKI   for kidney failure (95% CI)** | | **HR AKI vs no AKI   for death without kidney failure (95% CI)** | |
| --- | --- | --- | --- | --- | --- | --- |
| AKI vs no AKI | eGFR<30 | - | 1.11 | (0.79-1.55) | 1.80 | (1.20-2.71) |
| AKI vs no AKI | eGFR<30 | age, sex | 1.05 | (0.75-1.46) | 2.23 | (1.48-3.36) |
| AKI vs no AKI | eGFR<30 | age, sex, eGFR | 0.92 | (0.66-1.29) | 2.18 | (1.45-3.28) |
| AKI vs no AKI | eGFR<30 | age, sex, eGFR, proteinuria | 0.85 | (0.61-1.19) | 1.95 | (1.30-2.94) |
| AKI vs no AKI | eGFR<30 | age, sex, eGFR, proteinuria, renal diagnosis | 0.86 | (0.61-1.20) | 1.95 | (1.29-2.94) |
|  |  |  |  |  |  |  |
| AKI vs no AKI | eGFR≥30 | - | 2.85 | (1.82-4.45) | 2.24 | (1.44-3.50) |
| AKI vs no AKI | eGFR≥30 | age, sex | 2.80 | (1.79-4.38) | 2.46 | (1.58-3.84) |
| AKI vs no AKI | eGFR≥30 | age, sex, eGFR | 2.48 | (1.59-3.88) | 2.39 | (1.53-3.72) |
| AKI vs no AKI | eGFR≥30 | age, sex, eGFR, proteinuria | 2.39 | (1.53-3.73) | 2.10 | (1.34-3.27) |
| AKI vs no AKI | eGFR≥30 | age, sex, eGFR, proteinuria, renal diagnosis | 2.34 | (1.50-3.66) | 2.03 | (1.29-3.17) |
| *Note*: Age as linear term, eGFR^-2^, proteinuria in four KDIGO albuminuria categories of “severe”, “moderate”, “normal/mild” and “not tested”. AKI represents AKI occurring in a 2 year observation period prior to study follow up. eGFR and proteinuria status were determined on the last available sample at the end of the 2 year observation period.  Abbreviations: AKI, acute kidney injury; CI, confidence interval; eGFR, estimated glomerular filtration rate; HR, hazard ratio | | | | | | |
